# eRegQual—an electronic health registry with interactive checklists and clinical decision support for improving quality of antenatal care: study protocol for a cluster randomized trial

**DOI:** 10.1186/s13063-017-2386-5

**Published:** 2018-01-22

**Authors:** Mahima Venkateswaran, Kjersti Mørkrid, Buthaina Ghanem, Eatimad Abbas, Itimad Abuward, Mohammad Baniode, Ole Frithjof Norheim, J. Frederik Frøen

**Affiliations:** 1 Division for Health Services, Norwegian Institute of Public Health, PB 4404, Nydalen, N-0403 Oslo, Norway; 2 Centre for Intervention Science in Maternal and Child Health (CISMAC), University of Bergen, Bergen, Norway; 3 Palestinian National Institute of Public Health, World Health Organization, P.O. Box 4284, Al-Bireh, Palestine; 4 Department of Global Public Health and Primary Care, University of Bergen, Bergen, Norway

**Keywords:** Interactive checklists, Clinical decision support, Quality of care, Antenatal care, Maternal and newborn health, eHealth, Electronic registry, eRegistries, Health systems, Health surveillance

## Abstract

**Background:**

Health worker compliance with established best-practice clinical and public health guidelines may be enhanced by customized checklists of care and clinical decision support driven by point-of-care data entry into an electronic health registry. The public health system of Palestine is currently implementing a national electronic registry (eRegistry) for maternal and child health. This trial is embedded in the national implementation and aims to assess the effectiveness of the eRegistry’s interactive checklists and clinical decision support, compared with the existing paper based records, on improving the quality of care for pregnant women.

**Methods:**

This two-arm cluster randomized controlled trial is conducted in the West Bank, Palestine, and includes 120 clusters (primary healthcare clinics) with an average annual enrollment of 60 pregnancies. The intervention tool is the eRegistry’s interactive checklists and clinical decision support implemented within the District Health Information System 2 (DHIS2) Tracker software, developed and customized for the Palestinian context. The primary outcomes reflect the processes of essential interventions, namely timely and appropriate screening and management of: 1) anemia in pregnancy; 2) hypertension in pregnancy; 3) abnormal fetal growth; 4) and diabetes mellitus in pregnancy. The composite primary health outcome encompasses five conditions representing risk for the mother or baby that could have been detected or prevented by high-quality antenatal care: moderate or severe anemia at admission for labor; severe hypertension at admission for labor; malpresentation at delivery undetected during pregnancy; small for gestational age baby at delivery undetected during pregnancy; and large for gestational age baby at delivery. Primary analysis at the individual level taking the design effect of the clustering into account will be performed as intention-to-treat.

**Discussion:**

This trial, embedded in the national implementation of the eRegistry in Palestine, allows the assessment of process and health outcomes in a large-scale pragmatic setting. Findings will inform the use of interactive checklists and clinical decision support driven by point-of-care data entry into an eRegistry as a health systems-strengthening approach.

**Trial registration:**

ISRCTN trial registration number, ISRCTN18008445. Registered on 6 April 2017.

**Electronic supplementary material:**

The online version of this article (doi:10.1186/s13063-017-2386-5) contains supplementary material, which is available to authorized users.

## Background

Monitoring progress in universal health coverage requires reliable data that sufficiently depict the quality of health services and complexities of health systems [1]. The availability of relatively well-defined interventions and measurement indicators for reproductive, maternal, newborn and child health (RMNCH) provides a head start in monitoring and, ultimately, achieving universal health coverage within this sphere [2]. To enhance the quality of care in RMNCH in low- and middle-income settings, implementation of innovative health system interventions should be prioritized [3]. Checklists and clinical decision support for care providers are tools that can enhance quality of healthcare service delivery. Checklists support implementation of interventions and protocols at all levels of a health system among all care providers in a cadre, and have the ability to standardize decision-making throughout the health system [4–6]. The World Health Organization 29-item ‘Safe Childbirth Checklist’, designed to facilitate childbirth protocols, has been demonstrated to improve provision of safe practices by health workers and the quality of care [7, 8]. Interactive checklists on electronic platforms can provide decision and guideline support in response to structured data entry by care providers at the point-of-care (Table 1). Interactive checklists and clinical decision support, as compared to static checklists, have the advantage of offering individual case-based management suggestions [9]. When data entry is made online into a centralized electronic registry (eRegistry), the system not only supports the quality of care delivered by the individual care provider at one specific patient contact, but also supports the patient-centered continuity and quality of care along and across the health system [10]. The interactivity in the checklists, customized to health workers and their setting and level of health system, may form an important tenet for its effective use. Point-of-care support tools for health workers can help overcome frequent barriers to high-quality clinical care, such as the lack of accessible guidelines and validated management suggestions [11]. Interactive checklists and clinical decision support can potentially strengthen health workforce capacity and thus contribute to strengthening universal health coverage. Studies on electronic health (eHealth) and mobile health (mHealth) report good potential for such tools [12–14], and in the RMNCH field they may facilitate healthcare practice, health communication, and health education [15, 16]. Health systems may be made more effective, efficient, and accountable by exploiting opportunities of communications enabled by health information systems. However, robust research and evidence evaluating such interventions are scarce [17]. A systematic review of randomized controlled trials (RCTs) to study the effectiveness of any form of clinical decision support system linked to electronic health records showed a moderate effect on morbidity [18]. Most large RCTs for electronic health information systems, clinical decision support systems, and electronic registries have been conducted in high-income countries, with obviously different contextual considerations [19]. Implementing clinical decision support tools and electronic registries is resource-intensive, and substantial efforts are required in design, implementation, training, and support [20]. Considering this, there is a need for RCTs of the health systems to justify the extent of use of e- and mHealth solutions in low- and middle-income countries, particularly in primary healthcare where there is the most relevant demand for evidence [21].

**Table 1:** List of working definitions describing different components constituting intervention and the delivery platform

Term	Working definition
eRegistries	Electronic registries (eRegistries) are systems using information and communication technologies for the systematic longitudinal collection, storage, retrieval, analysis, and dissemination of uniform information on health determinants and outcomes of individual persons, to serve healthcare services, health surveillance, health education, knowledge, and research [10].
Interactive checklists	Interactive checklists are checklists delivered on electronic platforms. eRegistry’s interactive checklists integrate individualized decision support for daily clinical procedures, diagnosis, management, and referral routines in response to systematic point-of-care data entry by healthcare providers [10].
Clinical decision support system	An electronic system designed to aid directly in clinical decision making, in which characteristics of individual patients are used to generate case-specific assessments or recommendations that are then presented to clinicians for consideration [33].

### Palestinian context

Maternal and child health forms an important part of the healthcare system in Palestine. There are approximately 60,000 live births in the West Bank annually, and 99% are reported to be delivered in hospitals [22]. In 2014, the Palestinian Ministry of Health (MoH) operated 418 primary healthcare clinics (PHC) in the West Bank, and the majority of them provided antenatal care (ANC) services. PHC operated by the Palestinian MoH are classified from level 1 to 4 according to the healthcare services they provide. Level 1 PHC typically operate only once a week with a single healthcare provider, whereas level 4 PHC operate several days a week and with different health workers including specialists. ANC services are offered at all levels, but labor and delivery services are not provided in primary healthcare. Women have predefined PHC where they go for their ANC visits based on their residential address. According to the annual report published by the Palestinian MoH in 2016, adherence to at least one ANC visit is 98%, and to four visits 94%. About 45% of pregnant women in the West Bank receive ANC in PHC operated by the Palestinian MoH, with an average of 4.8 visits per woman. ANC is also provided by private clinics and the United Nations Relief and Works Agency for Palestine Refugees in the Near East (UNRWA). Comprehensive pregnancy risk management is provided at so-called high-risk clinics. There are 86 high-risk clinics in the West Bank and in 2015, 14% (*n* = 4415) of women receiving ANC in PHC operated by the Palestinian MoH were referred to a high-risk clinic for management. The Palestinian National Institute of Public Health (PNIPH) and the Palestinian MoH are currently rolling out a nationwide electronic registry (eRegistry) for maternal and child health. The implementation is undertaken in collaboration with and building on the eRegistries Initiative, tools, and implementation guidance, developed by the Norwegian Institute of Public Health in collaboration with the World Health Organization, the University of Oslo, and global RMNCH experts [10, 23]. The eRegistry for Palestine is developed in the District Health Information System 2 (DHIS2) tracker software, which is a flexible web-based open-source information system. All Palestinian residents have unique identifiers which makes it possible to track care and outcomes of women throughout pregnancy, childbirth, and the postpartum period, irrespective of the healthcare facility level. In this setting, currently operating only with paper case notes, we aim to assess the effectiveness of an eRegistry’s interactive checklists and clinical decision support on improving the quality of care for pregnant women.

### Objectives

#### Primary objectives

To estimate the effectiveness of the eRegistry’s interactive checklists and clinical decision support on: 1) improving the provision of timely and appropriate screening and management for important conditions during ANC; and 2) health outcomes for the mothers and the newborns.

#### Secondary objectives

To estimate the effectiveness of the eRegistry’s interactive checklists and clinical decision support on: 1) timely ANC visits; and 2) occurrence of stillbirths.

## Methods

### Trial design

This cluster randomized controlled trial (CRCT) is a superiority trial with two parallel arms (intervention and control). The unit of randomization is the individual PHC or, for the smallest units, clusters of two or three PHC. The randomization was undertaken by a statistician not otherwise involved in the trial.

### Study setting

The national public health system in Palestine currently uses paper-based files and reporting. An antenatal record is opened at the first ANC visit at the PHC. This record includes the personal ID number and name; socioeconomic information such as address, mother’s years of education, age at marriage and age at first pregnancy, monthly household income, and number of members in the household; obstetric information from the current and previous pregnancies such as parity, gestational age, last menstrual period, estimated date of delivery, and conception during breastfeeding; medical and surgical conditions; and family history of diseases (diabetes mellitus, hypertension, renal disease, etc.). Risk assessments are conducted at the first ANC visit, and women identified with certain risk factors are referred for further management in so-called high-risk clinics. Ultrasound scans during pregnancy are performed at the PHC. Monthly reports on aggregate data are sent from all PHC, high-risk clinics, and hospitals to the Palestinian MoH. Previous research of RMNCH services at different levels of the healthcare system in Palestine suggests considerable room for improvement [24]. Problems identified during stakeholder discussions as contributing to lower quality of care were sizable variations in the quality of care provided and ineffective ANC content despite frequent visits. Stakeholders in Palestine also identified several inefficiencies in the workflow in the PHC, due to redundant paperwork and reporting. Recommendations provided by Palestinian and international experts for the national health system include immediate action to support standardized practice guidelines, minimize inefficiencies, and promote better health information systems in RMNCH [24].

### Eligibility criteria

**Fig. 1:**
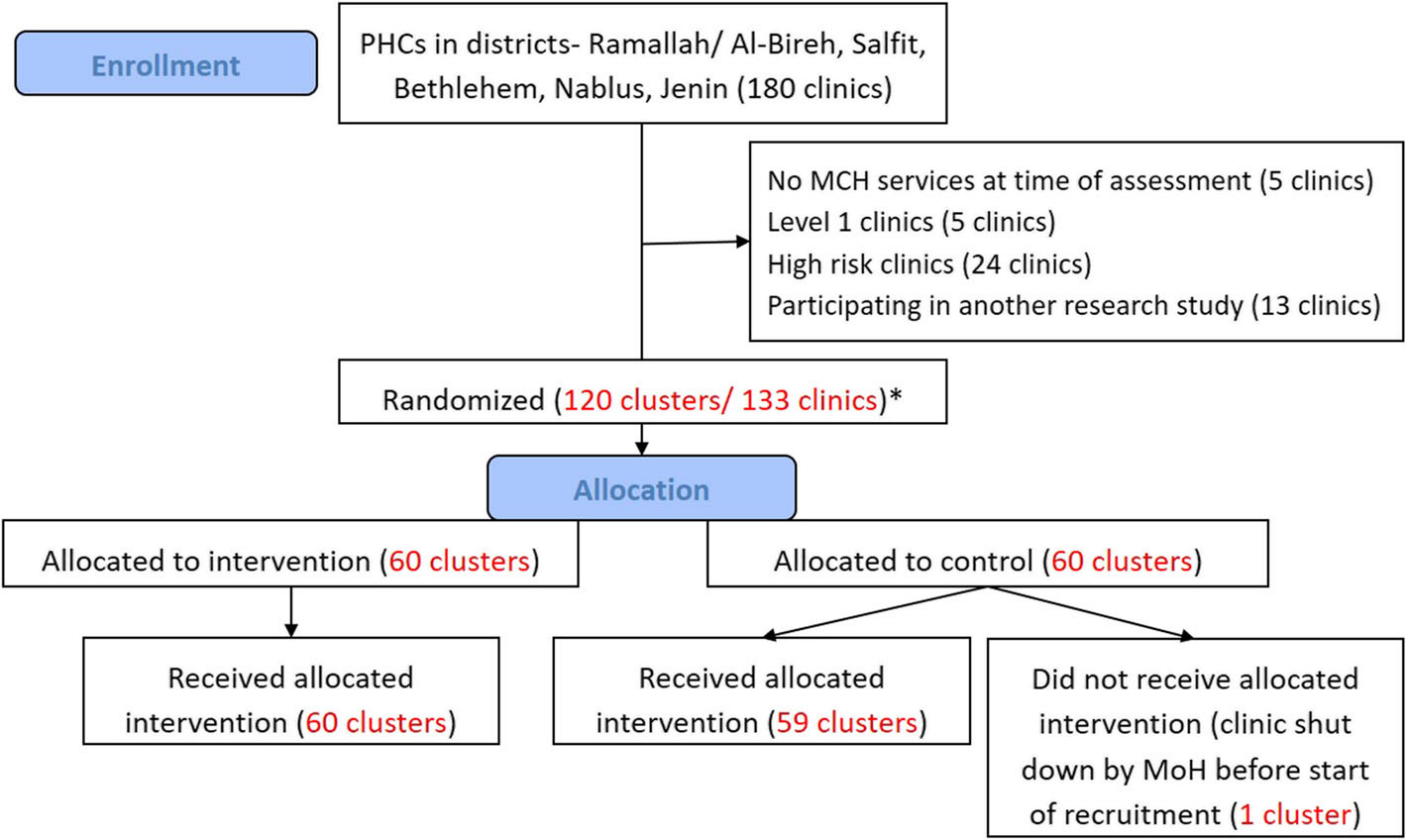
Flow diagram showing eligibility screening and recruitment of primary healthcare clinics (*PHC*). *We have grouped 25 small clinics in terms of annual number of newly enrolled preganancies (cluster size) to form 11 contiguous pairs and 1 contguous group of three clinics. The remaining PHC (*n* = 108) are individual clusters of their own, resulting in 120 clusters in total. *MCH *Maternal and Child Health, *MoH* Ministry of Health

The inclusion criteria were PHC located in Bethlehem, Jenin, Nablus, Ramallah/Al-Bireh, and Salfit governorates offering ANC services reporting to the Palestinian MoH, including PHC following the national guidelines and workflows but jointly operated by the Palestinian MoH and nongovernmental organizations (NGO). We excluded PHC defined as level 1 or with no pregnant women enrolled in 2013 (due to small cluster sizes), PHC with a high-risk clinic at the same location (because all referral PHC providing high-risk management must receive the eRegistry’s interactive checklists and clinical decision support to enable the continuity of care and maintain information flow for women from the intervention clusters), and PHC participating in another health system study addressing the quality of ANC. A total of 133 PHC were eligible for randomization (Fig. 1). These PHC enrolled 8061 (mean 60.6 and median 53.0) new pregnant women in 2013. All new enrollments (women visiting the PHC for ANC services for the first time in the current pregnancy) in the eligible PHC will be counted towards outcome measures of the study. As a health systems research study, there are no eligibility criteria related to individual women’s characteristics.

### Intervention

Care providers in the intervention PHC have received the eRegistry’s interactive checklists with clinical decision support. During the first 3–6 months, all intervention PHC had both paper and electronic files, as only new enrollments of pregnancies are included in the eRegistry. Care providers in the control PHC will continue using the current system of paper-based files throughout the trial period. In terms of the national implementation, the control PHC will receive the eRegistry at a later stage than the intervention PHC.

#### Intervention tool

**Fig. 2:**
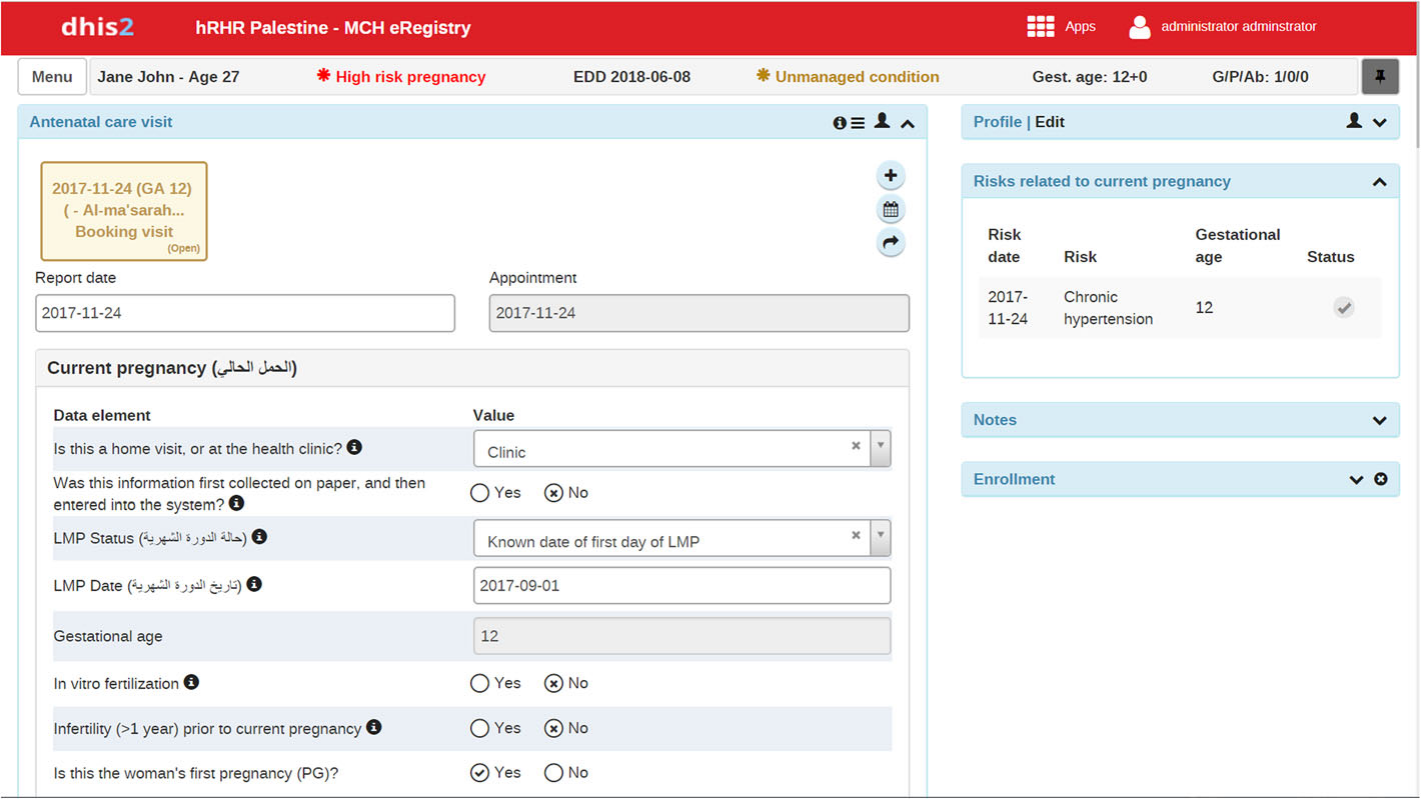
Intervention: illustration of interactive checklists. The top bar shows computed age, risk status, expected date of delivery (*EDD*), management status, gestational age at visit, and obstetric score

**Fig. 3:**
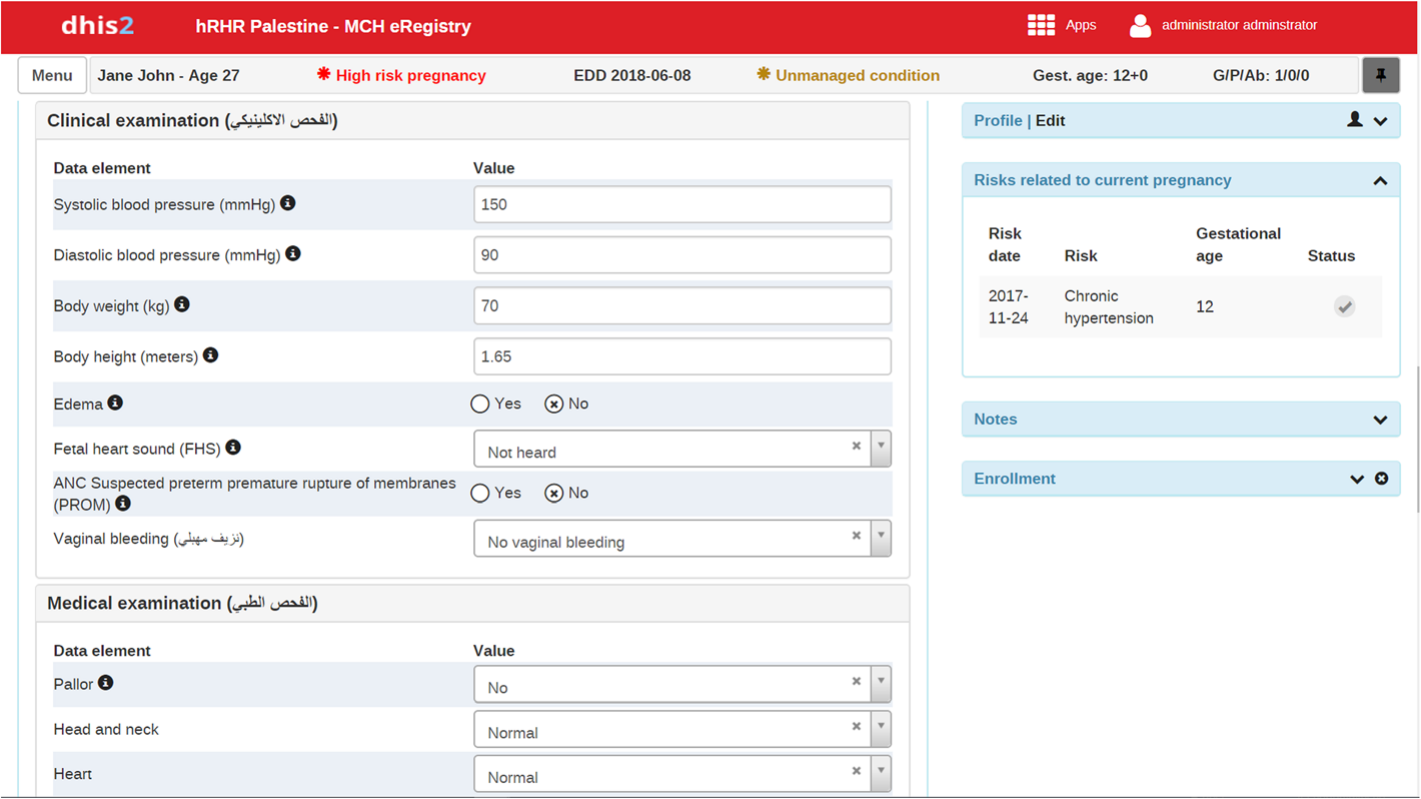
Intervention: illustration of clinical decision support. Clinical decision support shows chronic hypertension on the right panel in response to high blood pressure values at a gestational age of 12 weeks

**Fig. 4:**
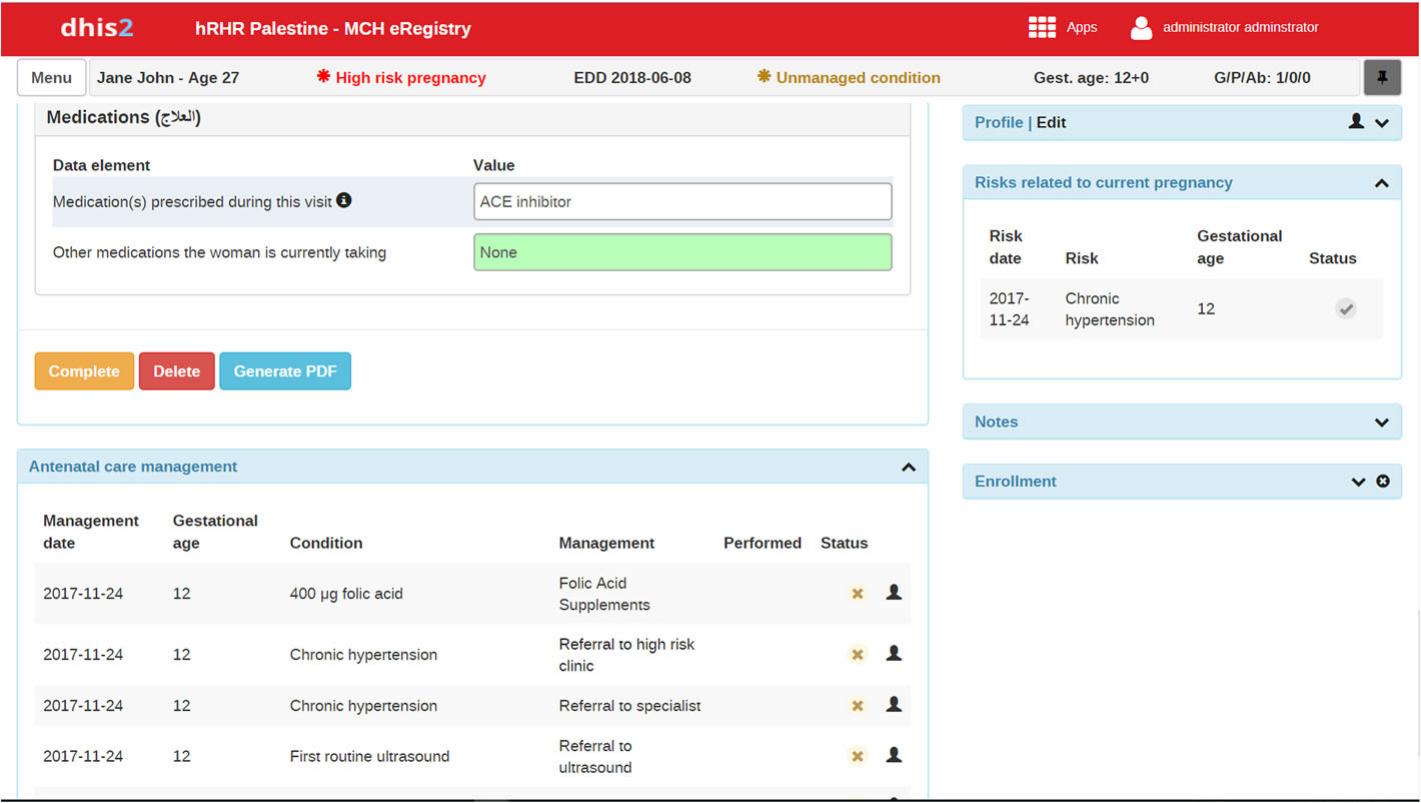
Intervention: illustration of clinical management reminders. The bottom panel shows routine management reminders according to the antenatal care visit (folic acid supplementation, first routine ultrasound) and management support for the identified risk condition (referral to high-risk clinic and specialist)

The intervention tool is the eRegistry’s interactive checklists and clinical decision support for ANC (Table 1). The interactive checklists and the current paper-based files include the same items and data points. The eRegistry allows for seamless incorporation of clinical workflow and guideline support in addition to reminders of daily clinical procedures and referrals (Table 1). All intervention PHC have been provided with desktop computers to be used by care providers in the consultation rooms. Care providers enter data on pregnant women into the eRegistry at the point-of-care (Table 1), and this data entry triggers the guideline-based decision support system (Figs. 2, 3 and 4). Each user has a unique username and password identifying their authorized access to the eRegistry in Palestine, with functionalities defined by their roles. The eRegistry is accessed by care providers using a Google Chrome browser. The full extent of the intervention is available at the demonstration eRegistry website which can be accessed through the link provided in reference [25].

#### Adherence

The eRegistry will fully replace the paper-based system. It is mandatory for use by care providers in the public health system in the intervention areas. Care providers in the intervention PHC have been trained on how to contact support staff in case of any problems with the software or infrastructure. Standard operating procedures (SOP) of the eRegistry in Palestine include routines for replacing malfunctioning computers and providing a back-up wireless internet solution within 24 h in case of internet disruption. The SOP also include measures for routine monitoring of use of the eRegistry and periodic data quality checks [26].

#### Concomitant care

The women seeking care in the intervention and control PHC will not receive any differential interference or concomitant care. The treatment guidelines were set by the Palestinian MoH, who were also responsible for the training and implementation of guidelines irrespective of allocation in this CRCT. The Palestinian MoH conducted a guidelines training session for intervention and control PHC in January 2016. The PNIPH, Palestinian MoH, and the Norwegian Institute of Public Health collaborate closely with the researchers of this CRCT, and have a common understanding of the importance of nonpreferential management of PHC. Any guideline change during the course of the CRCT will be applied to both the intervention and control PHC. The Palestinian MoH staff, referred to as maternal and child health supervisors that are typically nurses, carry out similar periodic supervision visits to all PHC in both arms of this CRCT.

### Outcomes

The focus of the outcome measures have been informed by national stakeholder consultations as representing key areas of quality concern in the Palestinian context, and to serve as sentinel outcomes for the overall quality of care. The adverse pregnancy outcomes reflect conditions that put the baby and/or mother at risk during labor or after delivery, and that could or should have been identified and/or managed prior to labor and delivery. The process (adherence) outcomes (Table 2) for the screening and management of the risk conditions and the corresponding adverse pregnancy outcomes (Table 3) will be assessed as primary outcomes. We have defined indicators to measure the process (adherence) outcomes (Additional file 1) based on the current guideline-based algorithms for management of conditions during ANC in Palestine (Additional file 2). Secondary outcomes are given in Table 4.

**Table 2:** Primary process (adherence) outcomes, source of data, measurement sequence, and definitions

Process (adherence) outcomes	Source of data	Measurement sequence	Definitions
Timely and appropriate screening and management of anemia during pregnancy	Case records data from the PHC in the eRegistry	Registrations continuously at point of care, data export to the trial monthly	Proportion of women attending ANC who receive: 1. anemia screening at booking, and 2. screening at any ANC visits at 24 and 36 weeks if no anemia detected, and 3. rescreening after 1 month if mild or moderate anemia is detected, and 4. referred to high-risk clinic if refractory mild or moderate anemia is detected, and 5. referred to hospital if severe anemia is detected.
Timely and appropriate screening and management of hypertension in pregnancy	Case records data from the PHC in the eRegistry	Registrations continuously at point of care, data export to the trial monthly	Proportion of women attending ANC who receive: 1. blood pressure measurement at booking visit, and 2. blood pressure measurement at every ANC visit, and 3. appropriate laboratory tests for mild hypertension, and 4. referred to high-risk clinic hospital for chronic or gestational hypertension, and 5. referred to hospital for hypertension with proteinuria or signs of eclampsia.
Timely and appropriate screening and management of abnormal fetal growth	Case records data from the PHC in the eRegistry	Registrations continuously at point of care, data export to the trial monthly	Proportion of women attending ANC who receive: 1. first fundal height measurement at 16–20 weeks, and 2. fetal growth monitoring at every ANC visit, and 3. referred to ultrasound if discrepancy between fundal height and gestational age, and 4. referred to high-risk clinic if ultrasound confirmed fetal growth restriction.
Timely and appropriate screening and management of diabetes in pregnancy	Case records data from the PHC in the eRegistry	Registrations continuously at point of care, data export to the trial monthly	Proportion of women attending ANC who receive: 1. diabetes screening at booking, and 2. screening with random blood sugar test at 24–28 weeks, and 3. referred to high-risk clinic if random blood sugar test or glucose challenge test ≥ 140 mg/dl.

*ANC* antenatal care, *eRegistry* electronic registry, *PHC* primary healthcare clinics

**Table 3:** Primary adverse pregnancy outcomes, source of data, measurement sequence, and definitions

Adverse pregnancy outcomes	Source of data	Measurement sequence	Definitions
Moderate or severe anemia at admission for labor	Hemoglobin admission data from hospitals in the eRegistry (public, private, and NGO hospitals)	Registrations continuously at point of care, data export to the trial monthly	Moderate anemia: hemoglobin greater than 7 and less than 9 g/dl; severe anemia: hemoglobin < 7 g/dl
Severe hypertension at admission for labor	Blood pressure admission data from hospitals (public, private, and NGO hospitals) in the eRegistry	Registrations continuously at point of care, data export to the trial monthly	Severe hypertension: systolic blood pressure ≥ 160 mmHg and/ or diastolic blood pressure ≥ 110 mmHg
Malpresentation at delivery undetected during pregnancy	Presentation at delivery data from hospitals (public, private, and NGO hospitals) in MCH registry	Registrations continuously at point of care, data export to the trial monthly	All non-cephalic presentations at or after 36 weeks of gestation and at labor
Small-for-gestational age baby at delivery undetected during pregnancy	Birth weight data from hospitals (public, private, and NGO hospitals) in the eRegistry	Registrations continuously at point of care, data export to the trial monthly	Small for gestational age: less than 2549 g [34]
Large for gestational age baby at delivery	Birth weight data from hospitals (public, private, and NGO hospitals) in the eRegistry	Registrations continuously at point of care, data export to the trial monthly	Large for gestational age: greater than 3980 g [34]

*eRegistry* electronic registry, *MCH *Maternal and Child Health, *NGO* nongovernmental organization

**Table 4:** Secondary outcomes, source of data, measurement sequence, and definitions

Secondary outcomes	Source of data	Measurement sequence	Definitions
Timely ANC visits	Case records data from the PHC in the eRegistry	Registrations continuously at point of care, data export to the trial monthly	Proportion of women attending ANC who receive timely ANC visits according to guidelines at: 1. booking visit 2. 16 weeks gestation 3. 18–22 weeks gestation 4. 24–28 weeks gestation 5. 32 weeks gestation 6. 36 weeks gestation
Timely and appropriate screening and management of malpresentation ≥ 36 weeks	Case records data from the PHC in the eRegistry	Registrations continuously at point of care, data export to the trial monthly	Proportion of women attending ANC who receive: 1. screening for fetal presentation at any visit ≥ 36 weeks, and 2. referred to hospital for non-cephalic presentation
Stillbirth	Stillbirth data from the hospitals (public, private, and NGO hospitals) in the eRegistry	Registrations continuously at point of care, data export to the trial monthly	Baby born with no signs of life at or after 28 weeks of gestation

*ANC* antenatal care, *eRegistry* electronic registry, *NGO* nongovernmental organization, *PHC* primary healthcare clinics

#### Effect on healthcare equity

The effects of the intervention on the equitable provision of healthcare across the outcome measures will be assessed. The data points that will be used to assess the effects on equity include: average monthly household incomes (less than 200; 200–900; 901–1824; 1825–3054; and > 3055 Israeli new Sheqel), mother’s years of education (<10 years, 10–13 years, > 13 years), age at marriage (less than 20; 21–25 years; 26–30; 31–35; 36–40; greater than 40 years), and age at first pregnancy (less than 20; 20–25; 26–30; 31–35; 36–40; greater than 40 years).

### Timeline

**Fig. 5:**
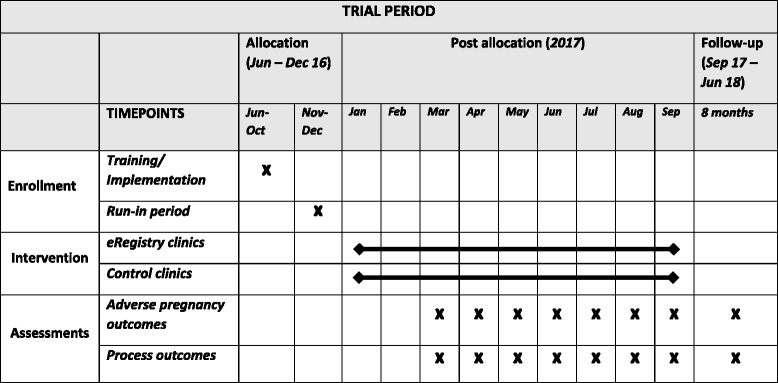
SPIRIT figure. Schedule of enrolment, intervention, and assessments. *eRegistry* electronic registry

The intervention sites have used the eRegistry exclusively for an average of 20 weeks (median of 16 weeks) prior to start of recruitment to ensure familiarity with the system while transitioning from paper files to the eRegistry (Fig. 5). The recruitment of new enrollments needed to reach the target sample size is expected to take approximately 8 months. There will be a follow-up period of another 8 months to capture adverse pregnancy outcomes for these new enrollments.

### Sample size

Sample size calculations for primary outcomes were performed in STATA (StataCorp. 2015, Stata Statistical Software: Release 14; College Station, TX, USA) “clustersampsi” using a 5% significance level and an assumed a priori intracluster correlation coefficient (ICC) of 0.01 for adverse pregnancy outcomes and 0.04 for the process outcomes [27]. Table 5 shows the estimated prevalence of primary outcomes for sample size calculations. We made sample size calculations to achieve a power of 90% based on an estimated average number of new pregnancies for 8 months in the study PHC. With 120 clusters recruiting for 8 months (with a minimum average cluster size of 44 in the year 2013), we will have more than 90% power to detect a relative 25–30% change in the composite adverse pregnancy outcome. With this sample size, we will have more than 90% power to detect a 15–25% change in the process primary outcomes. Effects of clustering and unequal cluster sizes have been accounted for in the sample size calculations (design effect = 1.75 for adverse pregnancy outcomes and 3.99 for process outcomes, and coefficient of variation of cluster size = 0.85) [28].

**Table 5:** Assumed prevalence for primary outcomes for calculations of sample size

Outcome measure	Control group prevalence
Adverse pregnancy outcomes: composite of any of the five conditions below, accounting for coexistence of conditions in 10% of women	0.145
1. Moderate or severe anemia at admission for labor	0.0225
2. Large-for-gestational age baby at delivery	0.054
3. Small-for-gestational age baby at delivery undetected during pregnancy	0.057
4. Malpresentation at delivery undetected during pregnancy	0.02
5. Severe hypertension at admission for labor	0.01
Process (adherence) outcomes	0.40–0.60

### Recruitment

The national implementation of the eRegistry is currently underway in Palestine. A total of 327 care providers from the intervention clinics were trained in the use of the eRegistry in batches of up to 20 participants per day of training. All care providers in the intervention PHC have had a day of introduction and 2 days of training in the use of the eRegistry. Each training session consisted of hands-on clinical case simulations focusing on data entry and documentation, clinical decision support functionalities, retrieving records, reporting routines, and general software maintenance. Initial group training sessions were followed up with visits to the PHC by the national implementation team to provide technical support to care providers [26]. No financial or nonfinancial incentives are provided to public health officers or care providers at the PHC.

### Allocation

PHC were randomized to either the control or the intervention group with a 1:1 allocation. We adopted an approach of stratified and covariate constrained randomizations. We stratified by district and constrained on: number of new enrollments of pregnancies per year (which reflects size and thus PHC level and days of operation per week); laboratory availability (which may affect care provider performance of screening tests); proportion of new enrollments above 40 years of age (which reflects general health and risk status); and proportion of primiparous women (which reflects risk status). A set of 10,000 randomization allocations were first generated. The 10% best and balanced allocations, with least differences between the two arms for the given covariates, were then identified. Finally, we randomly selected one of these allocations for the trial [29]. Statisticians at the Center for Intervention Science in Maternal and Child Health (CISMAC), University of Bergen, Norway, performed the randomization independently without any influence from the principal investigator or study staff. The list of participating clinics can be found in Additional file 3.

### Blinding

Participating PHC staff cannot be blinded to the allocation due to the nature of the intervention. During data collection for process outcomes, data collectors cannot be blinded to the allocation due to the nature of the intervention (paper collection versus electronic). The data collectors are, however, independent of the study team. They have received standardized training to collect a large number of routine indicators and data points for the eRegistry while being blinded for the outcome measures of this trial. The adverse pregnancy outcomes are collected independently by hospital staff using their routine health information system which is not linked to the eRegistry, effectively blinding hospital data collection to the allocation. Data analysts will be blinded to the allocation for all data management and computation of raw data for outcome measures, and for all analyses of primary and secondary outcomes. At the end of follow-up, each primary outcome will be analyzed separately. Dummy randomization variable codes will be generated for initial analyses of all primary outcomes by CISMAC (e.g., A and B, C and D, E and F, G and H, I and J), and the code will be provided as allocation groups (intervention versus control for each set) by CISMAC only after the completion of analyses.

### Data extraction methods

Care providers using the eRegistry or the paper records will continuously enter data during patient care. All data from completed paper records (after delivery of the baby) from the control PHC are routinely computerized by trained registry staff into the eRegistry every month. Double data extraction is carried out on approximately 10% of all the paper-based files for quality assurance purposes. Birth outcome data from public hospital maternity wards and delivery units are collected independently by hospital staff using their routine health information system which is not automatically linked to the eRegistry. These data are routinely exported from the hospital health information system’s national server every month, and merged into the eRegistry using the unique identifiers of the mothers. Data on births from all other hospitals with maternity wards and delivery units, including private hospitals and those run by NGOs, are collected by the Palestinian MoH staff using a standardized form on a monthly basis, and also merged into the eRegistry. For this CRCT we will only use anonymous data extracted from the eRegistry in accordance with the SOP of the Palestinian maternal and child health eRegistry for routine registry operations and use of data for research purposes [26].

### Data management

The data in the eRegistry will be managed in accordance with the governance structure approved by the Palestinian MoH [26]. Only predefined anonymous data needed for the outcomes will be provided to the principal investigator, the trial manager in Palestine, and to CISMAC for independent monitoring and safeguarding of the original dataset for analyses.

### Statistical methods

Data will be analyzed using STATA version 14 or later (StataCorp. 2015. Stata Statistical Software: Release 14*.* College Station, TX, USA). Primary analyses will be according to intention-to-treat comparing control and intervention groups using individual-level data taking the design effect of the clustering into account. In addition, we will undertake a per-protocol analysis. A baseline table with summary measures for the following background characteristics of participants will be reported: maternal age, parity, average monthly household income, education, medical history, obstetrical history, body mass index, blood pressure at booking visit, hemoglobin at booking visit, urine stick results for glucose at booking visit, birth weight, and fetal presentation at term. We will first undertake a cluster-level analysis for the primary and secondary outcomes. We will then perform analyses on individual-level data. All primary outcomes of the study will be measured as proportions. Comparisons of categorical variables will use generalized estimating equations (GEE) with a log link. We will use two-sided statistical tests and 95% confidence intervals for descriptive statistics and effectiveness estimates. In order to take into account the inherent clustering in our trial design, we will use random effects models. The effect of the intervention on the risk and prevalence of outcomes will be reported as relative (risk ratios (RR) and prevalence ratios (PR)) and absolute (risk difference and prevalence difference) measures of effect. Effectiveness will be calculated as 100 × (1 – RR) and 100 × (1 – PR) as appropriate. We will consider adjusting for confounders if there are baseline imbalances in the trial arms for variables that are strongly associated with the primary outcomes. We will perform complete case analyses and consider appropriate imputations for missing data. We will employ spider graphs in order to graphically display the effect of the intervention and whether a disproportionately large part of the effect can be ascribed to extreme effects in a few large clusters. In the rare event of protocol violations such as withdrawal of the eRegistry from an intervention PHC, data from these clusters will be excluded for women enrolled from the time of violation and onwards. All data on the pregnant women who maintain allocation, irrespective of from which cluster they receive care, will be included in the analyses. For those women that switch from intervention to control PHC, they will be analyzed as randomized in the primary, intention-to-treat analyses. In the per-protocol analysis, only data prior to switching clinics will be used and the rest will be treated as missing data. We will undertake instrumental variable analyses to better estimate the field efficacy of the intervention, in anticipation of possible nonadherence to the intervention [30]. For this analysis, the random allocation to the two arms of the CRCT will be the instrument. To enable this analysis, we will capture backdated data entry into the eRegistry from paper back-up (provided for use in cases of power outages and internet disruptions) to assess actual receipt of the intervention by the pregnant women. We will present additional analyses of quality of antenatal care measures in order to gain better understanding of the primary process (adherence) outcomes of the trial. These include: the proportion of women booked for care that both utilize and receive appropriate care; the probability of a woman affected by pregnancy complications to be successfully identified and managed; and provider-centric performance (the proportion of visits in which timely and appropriate care is provided).

### Data monitoring

This CRCT will be completed before the other clinics elsewhere in Palestine have completed their national implementation. PHC serving as controls for this CRCT will implement the eRegistry at the earliest opportunity that capacity allows. As there is insufficient manpower and infrastructure to introduce the intervention to all control sites earlier in case of overwhelming effectiveness, and no power of the research results to reverse the national decision to implement the eRegistry earlier than planned, no data monitoring committee will be established. Data management and monitoring will be performed in accordance with the SOP of the Palestinian maternal and child health eRegistry for routine registry operations [26].

### Harms

While the national implementation of the eRegistry in Palestine may create unexpected stress to the health system, in particular during the implementation and transition period, the study has no risks in itself. This CRCT only utilizes the moment of opportunity of an ongoing implementation to study a new health systems approach. Whether the eRegistry brings benefits or harms to the health system, the effect of the study itself is a measure of fairness since the sites receiving the new system first are chosen randomly within the districts.

### Audit and monitoring

CRCT monitoring is organized by CISMAC and will be independent of the researchers and sponsors. The first monitoring visit to assess CRCT readiness to begin recruitment took place on 28 and 29 September 2016. The second monitoring visit, during the trial, is expected within the first 6 months of inclusion.

### Confidentiality

Data confidentiality will be handled in accordance with the Palestinian MoH legal framework for maternal and child health electronic registries. This CRCT will only utilize anonymous registry data to enable the assessment of effectiveness, and only data that is pertaining to the outcome measures reflecting ANC provision. We will publish only aggregate data. We will not publish any data on individual clients, care providers, or identifiable clusters.

### Access to data

The data in the eRegistry belong to the Palestinian MoH and at no point or circumstance will the researchers have access to the entire registry or identifiable data of any kind.

### Dissemination plan

We followed the Standard Protocol Items: Recommendations for Intervention trials (SPIRIT) guidelines while writing this protocol (see Additional file 4). We will publish the results of the CRCT in peer-reviewed open-access journals and in presentations at scientific meetings and congresses. We will report the results in accordance with the Consolidating Standards of Reporting Trials (CONSORT) guidelines and the mHealth Evidence Reporting and Assessment (mERA) checklist. We will acknowledge any change in the study outcomes, study design, sample sizes, or significant administrative aspects that will impact the study’s nature when disseminating the findings. All authorship will be decided based on the recommendations of International Committee of Medical Journal editors. We will report the research findings to the Palestinian MoH directly. Summaries of the results and other relevant information will be published on the eRegistries website [23].

## Discussion

Electronic health information systems and programs are frequently tested in isolation, often as pilots or with less robust study designs [31]. This CRCT aims to contribute to filling this evidence gap. In order to study effectiveness of the intervention in the health system, we have designed a pragmatic CRCT. We have worked closely with the Palestinian MoH to plan the intervention and the timelines for the implementation of the eRegistry. All our communication strategies to the study clinics have always been through the Palestinian MoH via established channels within the health system in an attempt to keep monitoring of sites robust but as close to real-world settings as possible. We have striven to maintain generalizability of study findings by including a large representative sample of PHC and having no individual-level eligibility criteria. Data for outcome measures are derived from existing data collection pathways for the eRegistry in Palestine. The design and implementation of this CRCT places emphasis on the outcomes, as well as the feasibility of their measurement, in registry settings within the health system. Loss to follow-up is expected to be minimal since all our data sources are part of the eRegistry. We have designed outcome measures to directly reflect the effect of the intervention within the setting of an electronic registry, justifying the use of secondary data, where the process outcomes are based on the care providers’ own documentation of process, and health outcome measures are based on data collected independently from the care provider.

There are some limitations to the design of the CRCT. Pregnant women from intervention and control PHC are referred to the same high-risk clinics, where care providers use both paper records (for women referred from control PHC) and the eRegistry (for women referred from intervention PHC). This may lead to some contamination, which may underestimate the true effect of the intervention. About 5–10% of care providers in the study clinics work both in intervention and control PHC. This is another source of contamination that may potentially lead to effectiveness underestimation. Since the data for process outcomes is dependent on completeness of documentation by the care provider, there might be missing or incomplete data to inform some of the outcome indicators. We recognize that PHC location, activities, and staff are vulnerable to changes in public health planning and policy. We have tried to mitigate the risk of this affecting the study design by working closely with the Palestinian MoH and maintaining strong stakeholder involvement.

### Trial status

The CRCT started recruitment on 15 January 2017.

## Additional files

Additional file 1: Definitions and indicators for assessment of process/ adherence primary outcomes and secondary outcomes. (DOCX 19 kb)

Additional file 2: Management algorithms for outcome-related conditions during antenatal care in the public healthcare system in Palestine. (DOCX 189 kb)

Additional file 3: List of participating clinics by district and allocation. (DOCX 19 kb)

Additional file 4: Standard Protocol Items: Recommendations for Intervention trials (SPIRIT) 2013 checklist: recommended items to address in a clinical trial protocol and related documents. (DOCX 51 kb)

## Abbreviations

ANC: Antenatal care
CISMAC: Center for Intervention Science in Maternal and Child Health
CRCT: Cluster randomized controlled trial
DHIS2: District Health Information System 2
eHealth: Electronic health
eRegistry: Electronic registry
ICC: Intracluster correlation coefficient
mHealth: Mobile health
MoH: Ministry of Health
NGO: Nongovernmental organizations
PHC: Primary healthcare clinics
PNIPH: Palestinian National Institute of Public Health
RCT: Randomized controlled trial
RMNCH: Reproductive, maternal, newborn and child health
SOP: Standard operating procedures
