# Supplementary material for: eRegQual—an electronic health registry with interactive checklists and clinical decision support for improving quality of antenatal care: study protocol for a cluster randomized trial
Source: Trials. 2018 Jan 22;19:54. doi: 10.1186/s13063-017-2386-5 (PMC5778657; doi:10.1186/s13063-017-2386-5)
Supplement: Supplementary file 1 — Definitions and indicators for assessment of process/ adherence primary outcomes and secondary outcomes. (DOCX 19 kb) [file 13063_2017_2386_MOESM1_ESM.docx]

# Additional file 1: Definitions and indicators for assessment of process/ adherence primary outcomes and secondary outcomes

## Anemia in pregnancy

Definition for timely and appropriate screening and management for anemia in pregnancy:

- If ANC visit 1 (booking visit), anemia screening by Hb measurement- Screening done- 1, No screening- 0
- If no anemia in booking visit, and if ANC visit 23-30 weeks (and if no anemia at 23-30 weeks), at 34-37 weeks, anemia screening by Hb measurement- Screening done- 1, No screening- 0
- Any ANC visit if mild (10-10.9g/dl) or moderate anemia (7-9.9 g/dl), repeat hemoglobin after 3-5 weeks- Screening done- 1, No screening- 0
- Any ANC visit, previous mild anemia (10-10.9g/dl) or moderate anemia (7-9.9 g/dl), if no improvement in hemoglobin, referred to high risk clinic (unless already in high risk clinic)- Referred- 1, Not referred- 0
- Any ANC visit, Hb < 7 g/ dl, referred to hospital- Referred- 1, Not referred- 0

| Indicator | Numerator | Denominator | Data extraction syntax |
| --- | --- | --- | --- |
| Measures of routine screening | | | |
| Proportion of women attending ANC screened for anemia at booking | Number of women screened at booking | All women attending booking visit | If ANC visit 1 (booking visit), anemia screening |
| Proportion of women attending ANC with no anemia at booking screened at 24-28, 36 W | Number of women screened at 24-28, 36W | Women with no anemia in previous screening(s) | If ANC visit 23- 30 weeks, 34- 37 weeks, no anemia at previous visit, Hb measurement |
| Measures of risk management | | | |
| Proportion of women with mild/moderate anemia with repeat hemoglobin after 1 month of treatment* | Number of women with mild and moderate anemia with repeat hemoglobin after 1 month | Women with mild and moderate anemia | Any ANC visit, if mild anemia (10-10.9g/dl) or moderate anemia (7-9.9 g/dl), repeat hemoglobin after 3-5 weeks |
| Proportion of women with mild/moderate anemia referred appropriately | Number of women with mild/moderate anemia referred appropriately | Women with mild and moderate anemia | Any ANC visit, previous mild anemia (10-10.9g/dl) or moderate anemia (7-9.9 g/dl), if no improvement in hemoglobin, referred to high risk clinic |
| Proportion of women detected with severe anemia and referred appropriately | Number of women referred for severe anemia | Women with severe anemia | Any ANC visit, Hb < 7 g/ dl, referred to hospital |

## Hypertension in pregnancy

- If ANC visit 1 (booking visit), hypertension screening by blood pressure (BP) measurement- Screening done- 1, No screening- 0
- Any ANC visit (systolic BP < 140 mm Hg AND diastolic BP < 90 mm Hg), blood pressure measurement- Screening done- 1, no screening- 0
- If <20 weeks gestation and systolic BP ≥ 140 mm Hg and/or diastolic BP ≥ 90 mm Hg, referred for chronic hypertension- Referred- 1, not referred- 0
- If mild gestational hypertension (systolic BP 140–149 mmHg, diastolic BP 90–99 mmHg), urine test for protein done- Screening done- 1, no screening- 0
- If moderate (systolic BP 150–159 mmHg, diastolic BP 100–109 mmHg) or severe gestational hypertension (systolic BP ≥ 160 mmHg, diastolic BP ≥110 mmHg), referred to hospital- Referred- 1, not referred- 0
- If any ANC visit, chronic, mild, moderate or severe gestational hypertension with proteinuria, refer to hospital- Referred- 1, not referred- 0
- If any ANC visit, signs of eclampsia, referred to hospital- Referred- 1, not referred- 0

| Indicator | Numerator | Denominator | Data extraction syntax |
| --- | --- | --- | --- |
| Measures of routine screening | | | |
| Proportion of women attending ANC screened for hypertension at booking | Number of women screened at booking | All women attending booking visit | If ANC visit 1 (booking visit), hypertension screening |
| Proportion of women attending ANC with BP measurement at every ANC visit | Number of women screened at every visit | All women attending ANC | If any ANC visit, BP measurement |
| Measures of risk management | | | |
| Proportion of women with chronic hypertension referred appropriately | Number of women referred for preexisting hypertension | Women with preexisting hypertension | If ANC visit <20 weeks, systolic BP ≥ 140 mm Hg and/or diastolic BP ≥ 90 mm Hg, referred for preexisting hypertension |
| Proportion of women with mild gestational hypertension screened with urine protein | Number of women with mild gestational hypertension screened with urine protein | Women with mild gestational hypertension | Any ANC visit, mild gestational hypertension, screened with urine protein |
| Proportion of women with moderate or severe gestational hypertension referred appropriately | Number of women referred for moderate or severe gestational hypertension | Women with moderate or severe gestational hypertension | Any ANC visit, moderate or severe gestational hypertension, referred appropriately |
| Proportion of women with hypertension and proteinuria referred appropriately | Number of women with hypertension in pregnancy and proteinuria referred appropriately | Women with hypertension and proteinuria | Any ANC visit, if proteinuria with hypertension, referred appropriately |
| Proportion of women with eclampsia referred appropriately | Number of women referred for signs of eclampsia | Women with signs of eclampsia | If any ANC visit, signs of eclampsia, referred appropriately |

## Malpresentation at term

Definition for timely and appropriate screening and management for malpresentation at term:

- If any ANC visit ≥ 36 W, presentation checked- screened-1, not screened-0
- If any ANC visit ≥ 36 W, referred to ultrasound for malpresentation- Ultrasound done-1, Not done-0
- If any ANC visit ≥ 36 W, referred to hospital for malpresentation- Referred-1, not referred-0

| Indicator | Numerator | Denominator | Data extraction syntax |
| --- | --- | --- | --- |
| Measure of routine screening | | | |
| Proportion of women attending ANC who were screened for malpresentation ≥ 36 W of gestation | Number of women screened for malpresentation | All women attending ANC | If any ANC visit ≥ 36 W, presentation checked |
| Measures of risk management | | | |
| Proportion of women identified with malpresentation referred for ultrasound | Number of women referred to ultrasound for malpresentation | Women identified with malpresentation | If any ANC visit ≥ 36 W, referred to ultrasound for malpresentation |
| Proportion of women detected and referred appropriately for malpresentation | Number of women referred for malpresentation | Women detected with malpresentation | If any ANC visit ≥ 36 W, referred to hospital for malpresentation |

## Fetal growth monitoring

Definition for timely fetal growth monitoring and abnormal management for abnormal fetal growth:

- If every ANC visit at and after16-20 weeks until term, symphysiofundal height measurement- Measured- 1, Not measured- 0
- If any ANC visit > 16 weeks, referred to ultrasound examination for discrepancy- ultrasound done- 1, ultrasound not done- 0

If any ANC visit > 16 weeks, referred for discrepancy of fundal height- referred- 1, not referred- 0

| Indicator | Numerator | Denominator | Data extraction syntax |
| --- | --- | --- | --- |
| Measures of routine screening | | | |
| Proportion of women attending ANC with SFH measured at 16-20 weeks | Number of women whose SFH is measured at 16-20 weeks | All women attending antenatal care | If ANC visit 16-20 weeks, symphysiofundal height measurement |
| Proportion of women attending ANC with fetal growth monitoring at every visit after 16- 20 weeks | Number of women with fetal growth monitoring every visit after 16- 20 weeks | All women attending antenatal care | If any ANC visit 16- 20 weeks, fetal growth is monitored |
| Measures of risk management | | | |
| Proportion of women with a discrepancy referred appropriately for ultrasound examination | Number of women referred for discrepancy of fundal height to ultrasound | Women with discrepancy | If any ANC visit > 16 weeks, referred to ultrasound examination for discrepancy |
| Proportion of women with ultrasound confirmed small/ large for gestational age referred appropriately | Number of women referred for suspected small for gestational age fetus | Women detected with small for gestational age fetus | If any ANC visit > 16 weeks, referred for discrepancy of fundal height |

## Diabetes mellitus in pregnancy

Definition for timely and appropriate screening and management for diabetes mellitus in pregnancy:

- If ANC1 (booking visit), screening for glucose in urine- Screening done- 1, No screening- 0
- If ANC1 (booking visit), if glucose in urine positive, random blood sugar test- Screening done- 1, No screening- 0
- If ANC1 (booking visit), if random blood sugar test positive (RBS ≥ 140 mg/dl), referred to high risk clinic- Referred- 1, Not referred- 0
- If ANC visit 23-31 weeks, screening with Random blood sugar- Screening done- 1, No screening- 0
- If ANC visit 23-31 weeks, and if random blood sugar (≥ 140mg/dL), referred to high risk clinic- Referred- 1, Not referred- 0
- If ANC visit 23-31 weeks, and if random blood sugar (≥ 105-140mg/dl), glucose challenge test- Screening done- 1, No screening- 0
- Any ANC visit >23 weeks, if non Fasting: 1 hour 50 g Glucose Challenge Test positive (blood sugar ≥ 140 mg/dL) referred for gestational diabetes mellitus to high risk clinic- Referred-1, Not referred- 0

| Indicator | Numerator | Denominator | Data extraction syntax |
| --- | --- | --- | --- |
| Measures of routine screening | | | |
| Proportion of women attending ANC screened for glucose in urine at booking | Number of women screened for glucose in urine | All women attending antenatal care (booking visit) | If ANC1 (booking visit), screening for glucose in urine |
| Proportion of women attending ANC screened with random blood sugar at 24-28 weeks | Number of women screened with random blood sugar at 24-28 weeks | All women attending antenatal care | If ANC visit 23-31 weeks, screening with Random blood sugar |
| Measures of risk management | | | |
| Proportion of women with positive urine glucose stick who get random blood sugar test at booking visit | Number of women with positive urine glucose stick in urine and random blood sugar test at booking visit | Women with positive glucose stick | If ANC1 (booking visit), if glucose in urine positive, random blood sugar test |
| Proportion of women with positive random blood sugar test referred to high risk clinic | Number of women referred to high risk clinic for positive random blood sugar | Women with positive random blood sugar | If ANC1 (booking visit), if random blood sugar test positive, referred to high risk clinic |
| Proportion of women screened appropriately with glucose challenge test | Number of women with random blood sugar 105-140 mg/dl getting glucose challenge test | Women screened with random blood sugar at 24-28 weeks | If ANC visit 23-31 weeks, and if random blood sugar (≥ 105-140mg/dl), glucose challenge test |
| Proportion of women detected with gestational diabetes and referred appropriately | Number of women with referred for gestational diabetes | Women detected with gestational diabetes | Any ANC visit >23 weeks, referred for gestational diabetes mellitus to high risk clinic |
