# Supplementary material for: eRegQual—an electronic health registry with interactive checklists and clinical decision support for improving quality of antenatal care: study protocol for a cluster randomized trial
Source: Trials. 2018 Jan 22;19:54. doi: 10.1186/s13063-017-2386-5 (PMC5778657; doi:10.1186/s13063-017-2386-5)

# Additional file 2: Management algorithms for outcome-related conditions during antenatal care in the public healthcare system in Palestine

#
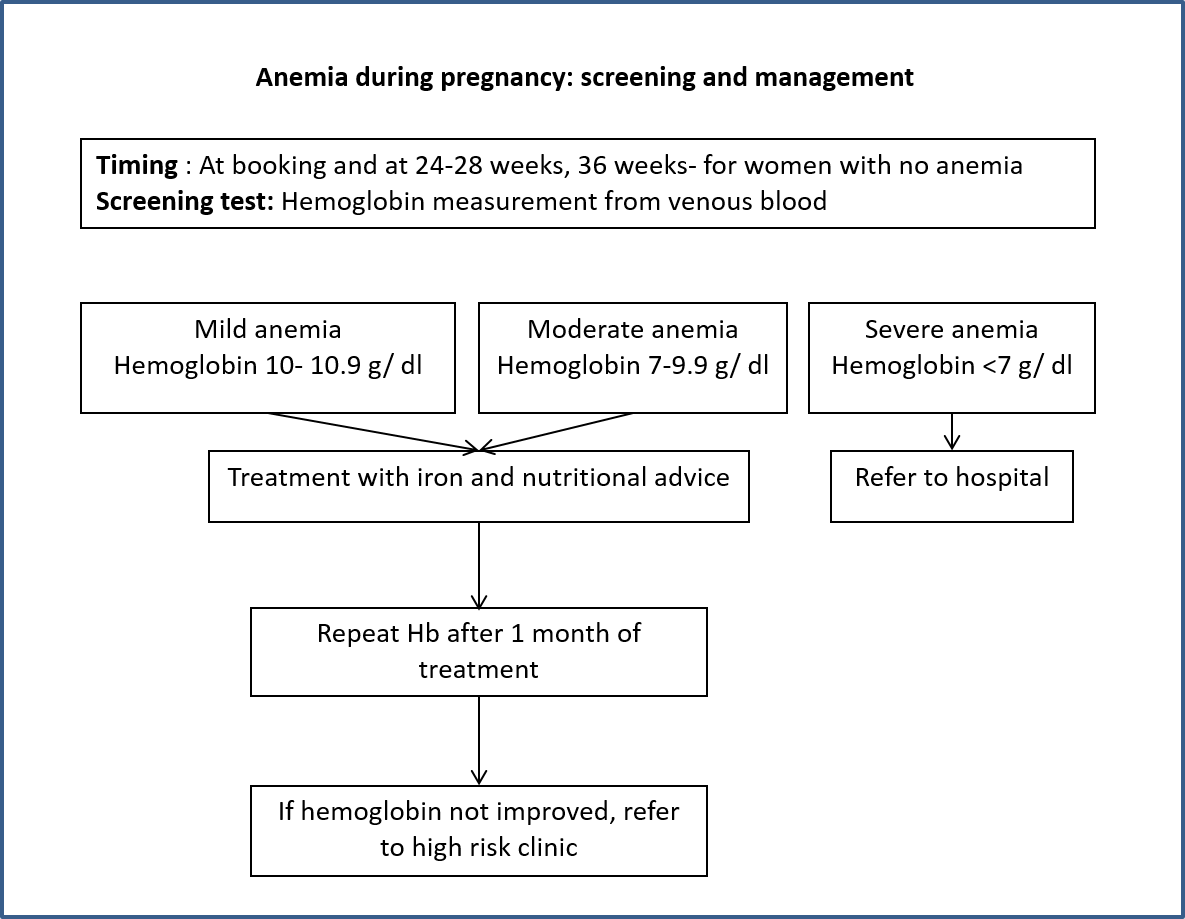


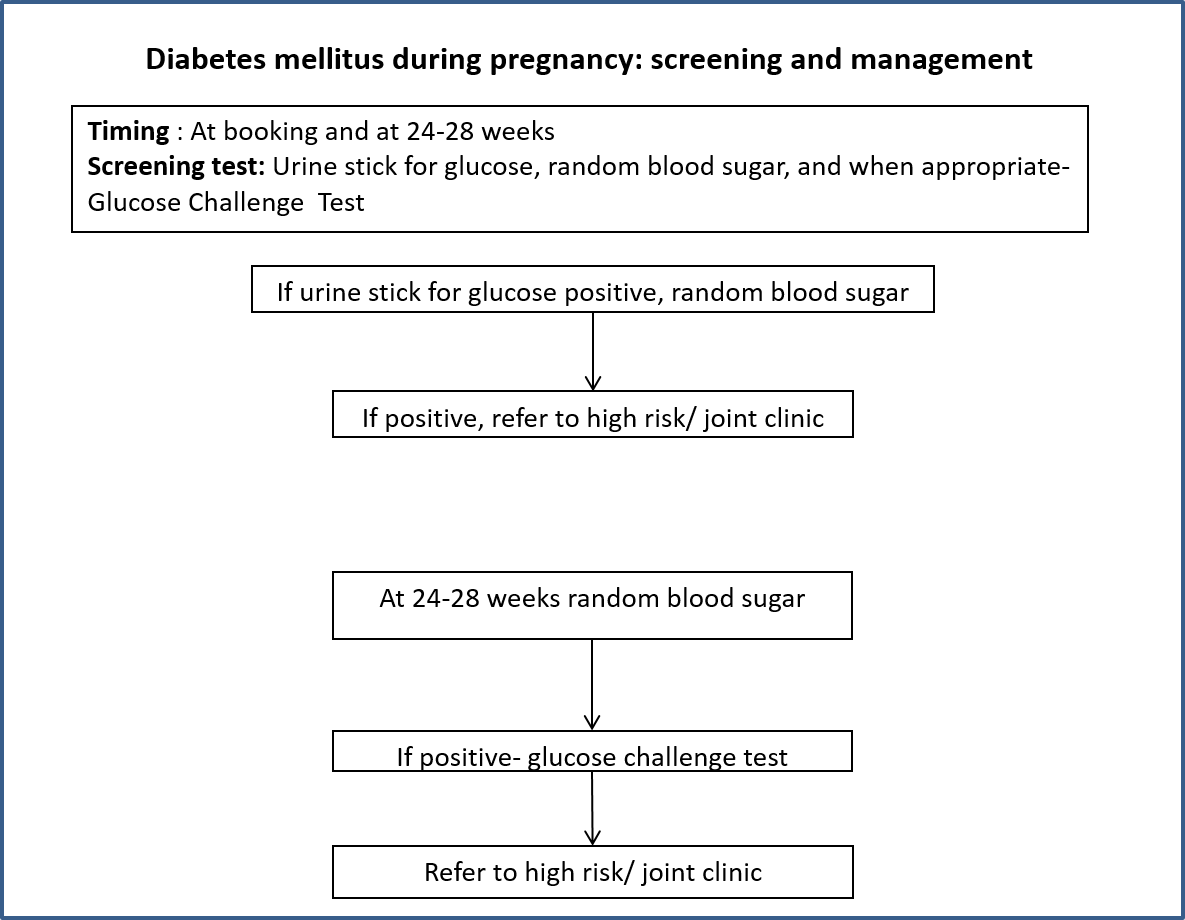


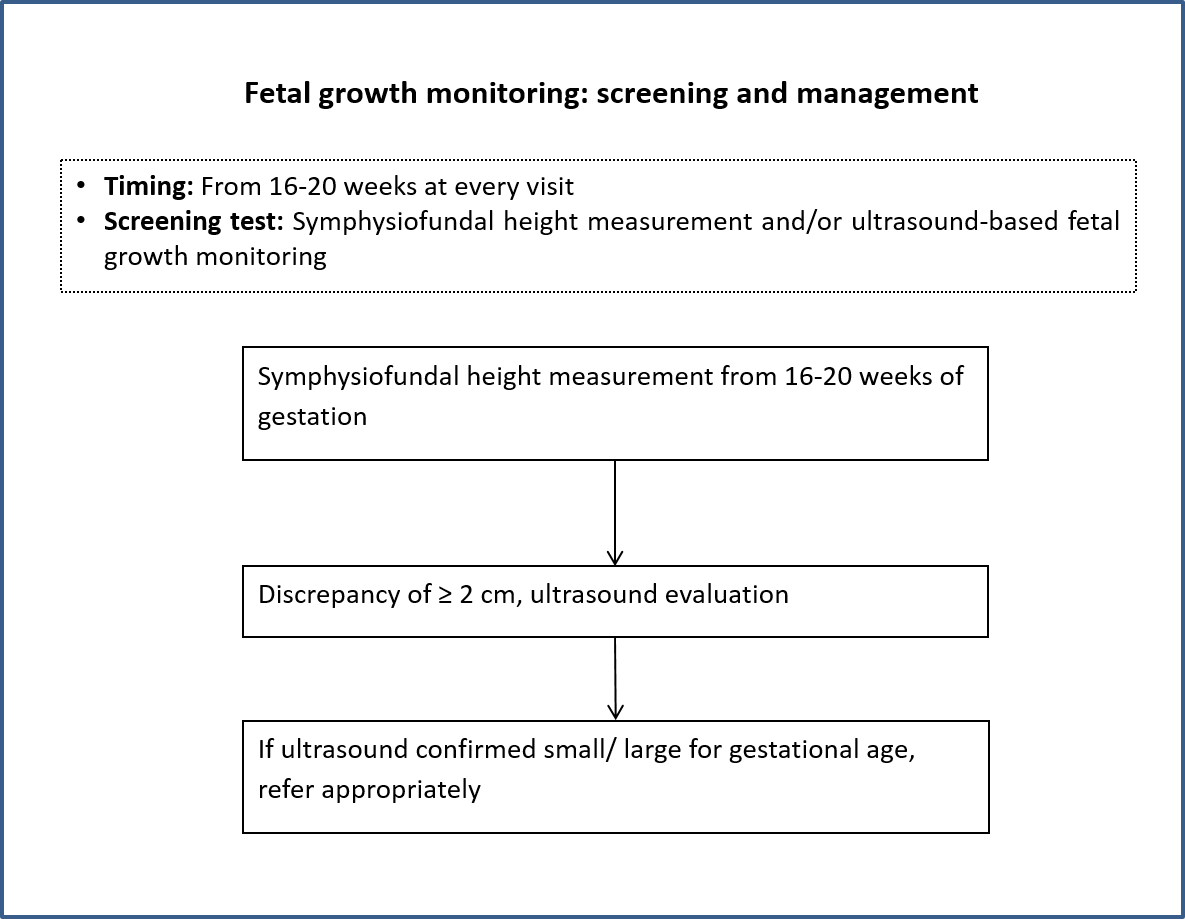


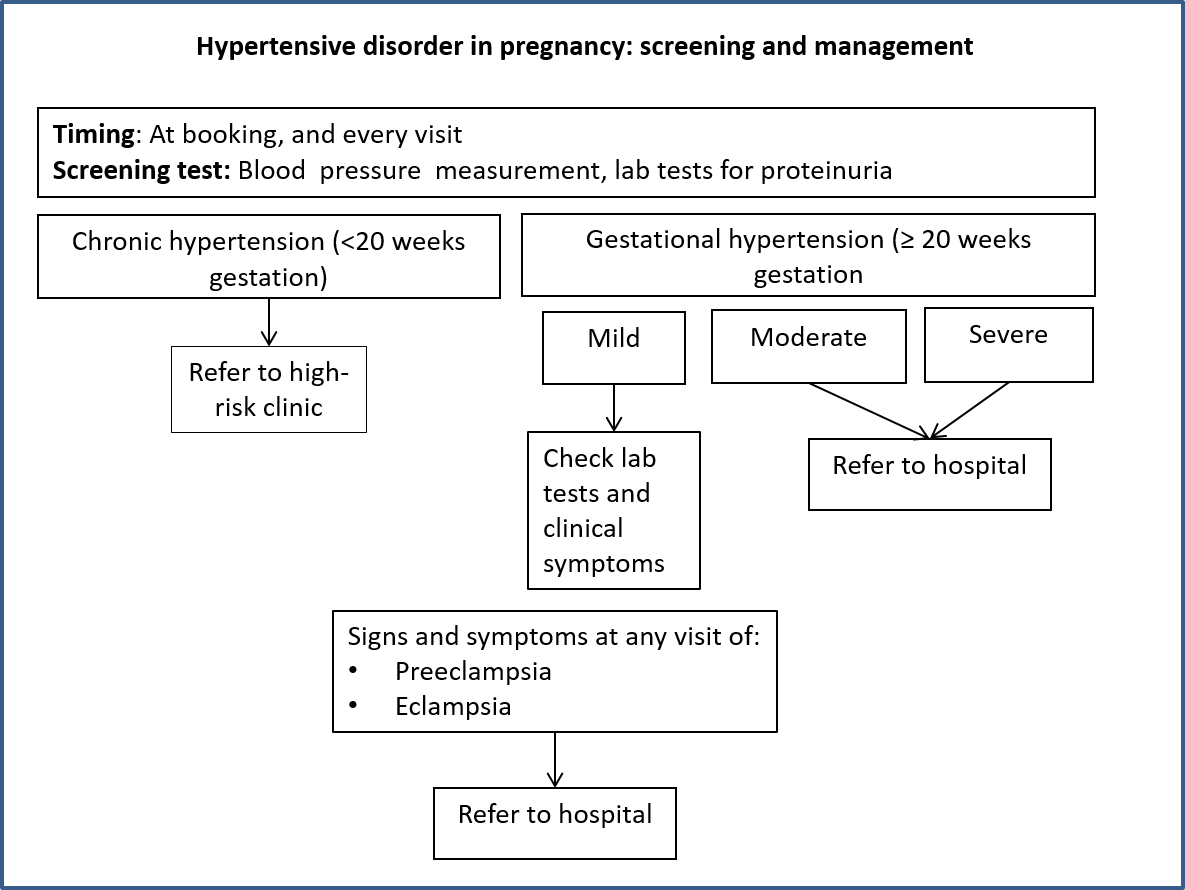


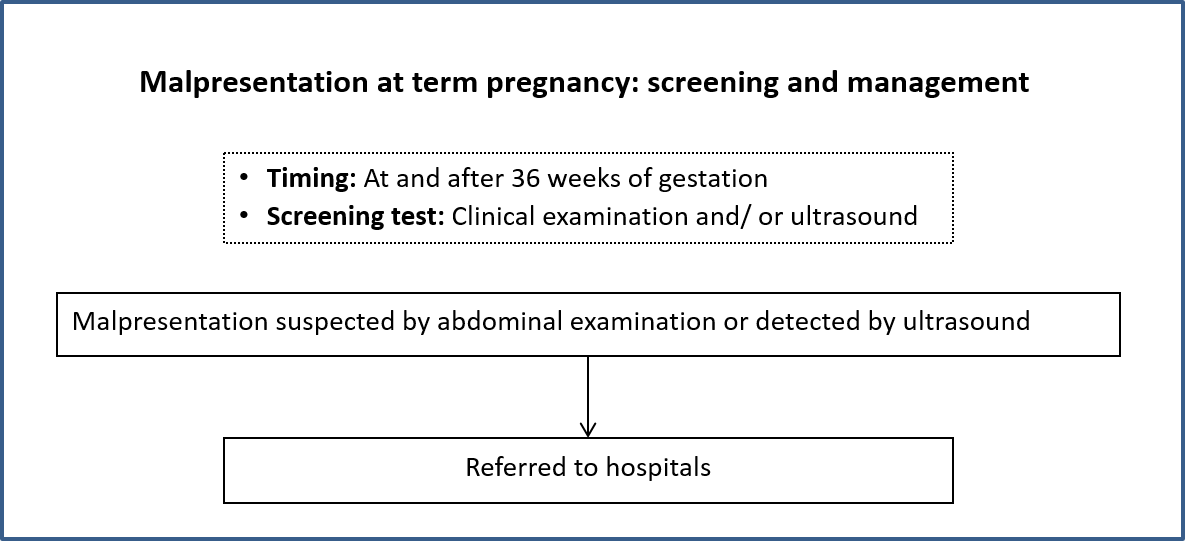

Supplement: Supplementary file 2 — Management algorithms for outcome-related conditions during antenatal care in the public healthcare system in Palestine. (DOCX 189 kb) [file 13063_2017_2386_MOESM2_ESM.docx]
