# Supplementary material for: eRegQual—an electronic health registry with interactive checklists and clinical decision support for improving quality of antenatal care: study protocol for a cluster randomized trial
Source: Trials. 2018 Jan 22;19:54. doi: 10.1186/s13063-017-2386-5 (PMC5778657; doi:10.1186/s13063-017-2386-5)
Supplement: Supplementary file 3 — List of participating clinics by district and allocation. (DOCX 19 kb) [file 13063_2017_2386_MOESM3_ESM.docx]

# Additional file 4: List of participating clinics by district and allocation

| S.no. | Name of clinic | District | Allocation |
| --- | --- | --- | --- |
|  | **Bateer** | Bethlehem | Control |
|  | **Beit Fjar** | Bethlehem | Control |
|  | **Beit Jala** | Bethlehem | Control |
|  | **Harmalah** | Bethlehem | Control |
|  | **Hosan** | Bethlehem | Control |
|  | **Joret Al-shamah** | Bethlehem | Control |
|  | **Tqoo'** | Bethlehem | Control |
|  | **Al-jadedah** | Jenin | Control |
|  | **Al-jalameh** | Jenin | Control |
|  | **Al-mghayer** | Jenin | Control |
|  | **Anza** | Jenin | Control |
|  | **Barta'h** | Jenin | Control |
|  | **Borqeen** | Jenin | Control |
|  | **Deir Abu D'eef** | Jenin | Control |
|  | **Fahmeh** | Jenin | Control |
|  | **Faqqua'** | Jenin | Control |
|  | **Jalboon** | Jenin | Control |
|  | **Kafr Dan** | Jenin | Control |
|  | **Kafr Kood** | Jenin | Control |
|  | **Rumaneh** | Jenin | Control |
|  | **Serees** | Jenin | Control |
|  | **Silat al-hartheyeh** | Jenin | Control |
|  | **Tura** | Jenin | Control |
|  | **Zboba** | Jenin | Control |
|  | **Al-baldeh Alqademah*** | Nablus* | Control* |
|  | **Al-nsaryeh** | Nablus | Control |
|  | **Aqraba** | Nablus | Control |
|  | **Asira al-Shamaliya'** | Nablus | Control |
|  | **Azmoot** | Nablus | Control |
|  | **Beit Umareen** | Nablus | Control |
|  | **Deir Alhatab** | Nablus | Control |
|  | **Deir Sharaf** | Nablus | Control |
|  | **Enabous** | Nablus | Control |
|  | **Oreef** | Nablus | Control |
|  | **Qusarh** | Nablus | Control |
|  | **Qusin** | Nablus | Control |
|  | **Sarah** | Nablus | Control |
|  | **Yetma** | Nablus | Control |
|  | **Al-Mazra' Al-gharbeieh** | Ramallah & Al-Bireh | Control |
|  | **Al-Midyah** | Ramallah & Al-Bireh | Control |
|  | **Arorah** | Ramallah & Al-Bireh | Control |
|  | **Beit liqia** | Ramallah & Al-Bireh | Control |
|  | **Beit Ur Altahta** | Ramallah & Al-Bireh | Control |
|  | **Birzeit** | Ramallah & Al-Bireh | Control |
|  | **Burqa** | Ramallah & Al-Bireh | Control |
|  | **Deir Abu Mash'al** | Ramallah & Al-Bireh | Control |
|  | **Deir Dibwan** | Ramallah & Al-Bireh | Control |
|  | **Deir Ibzee'** | Ramallah & Al-Bireh | Control |
|  | **Deir qdees** | Ramallah & Al-Bireh | Control |
|  | **Dura al Qarea'** | Ramallah & Al-Bireh | Control |
|  | **Etarah** | Ramallah & Al-Bireh | Control |
|  | **Kafr Malik** | Ramallah & Al-Bireh | Control |
|  | **kobar** | Ramallah & Al-Bireh | Control |
|  | **Qebiah** | Ramallah & Al-Bireh | Control |
|  | **Rantees** | Ramallah & Al-Bireh | Control |
|  | **Ras karkr** | Ramallah & Al-Bireh | Control |
|  | **Selwad** | Ramallah & Al-Bireh | Control |
|  | **Shebteen** | Ramallah & Al-Bireh | Control |
|  | **Al-zawyeh** | Salfit | Control |
|  | **Bruqin** | Salfit | Control |
|  | **Deir Istiya** | Salfit | Control |
|  | **Deir Ballut** | Salfit | Control |
|  | **Hares** | Salfit | Control |
|  | **Masha** | Salfit | Control |
|  | **Rafat** | Salfit | Control |
|  | **Wadi foqeen** | Bethlehem | Intervention |
|  | **Beit Sahoor** | Bethlehem | Intervention |
|  | **Al-ma'sarah** | Bethlehem | Intervention |
|  | **Al-shwawreh** | Bethlehem | Intervention |
|  | **Al-khader** | Bethlehem | Intervention |
|  | **Al-eabedieh** | Bethlehem | Intervention |
|  | **Al-zbabdeh** | Jenin | Intervention |
|  | **Marj Bani Amer/ Beit Qad** | Jenin | Intervention |
|  | **Al-hashmeyeh** | Jenin | Intervention |
|  | **Al-ramah** | Jenin | Intervention |
|  | **Kferet** | Jenin | Intervention |
|  | **Al-atarah** | Jenin | Intervention |
|  | **Al-Taibeh** | Jenin | Intervention |
|  | **Aneen** | Jenin | Intervention |
|  | **Raba** | Jenin | Intervention |
|  | **Jalqamoos** | Jenin | Intervention |
|  | **Al-Fandaqumiya** | Jenin | Intervention |
|  | **Mirka** | Jenin | Intervention |
|  | **Misilyah** | Jenin | Intervention |
|  | **AL-a'rakah** | Jenin | Intervention |
|  | **Kufr Raa'e** | Jenin | Intervention |
|  | **Meithalun** | Jenin | Intervention |
|  | **Al-naqorah** | Nablus | Intervention |
|  | **Beit Eba** | Nablus | Intervention |
|  | **Salem** | Nablus | Intervention |
|  | **Bazria** | Nablus | Intervention |
|  | **Rojeeb** | Nablus | Intervention |
|  | **Borqah** | Nablus | Intervention |
|  | **Talfeet** | Nablus | Intervention |
|  | **Awarta** | Nablus | Intervention |
|  | **Tel** | Nablus | Intervention |
|  | **Doma** | Nablus | Intervention |
|  | **Ras Al-een** | Nablus | Intervention |
|  | **Beita** | Nablus | Intervention |
|  | **Qabalan** | Nablus | Intervention |
|  | **Al-janyeh** | Ramallah & Al-Bireh | Intervention |
|  | **Beiteen** | Ramallah & Al-Bireh | Intervention |
|  | **Deir Netham** | Ramallah & Al-Bireh | Intervention |
|  | **Ein Yabrud** | Ramallah & Al-Bireh | Intervention |
|  | **Safa** | Ramallah & Al-Bireh | Intervention |
|  | **Deir Al-sudan** | Ramallah & Al-Bireh | Intervention |
|  | **Mazare' Alnobani** | Ramallah & Al-Bireh | Intervention |
|  | **Emsafa** | Ramallah & Al-Bireh | Intervention |
|  | **Al-tereh** | Ramallah & Al-Bireh | Intervention |
|  | **Bettello** | Ramallah & Al-Bireh | Intervention |
|  | **Kufr Ein** | Ramallah & Al-Bireh | Intervention |
|  | **Kharbatha Bani Hareth** | Ramallah & Al-Bireh | Intervention |
|  | **Rammun** | Ramallah & Al-Bireh | Intervention |
|  | **Bill'in** | Ramallah & Al-Bireh | Intervention |
|  | **Ebween** | Ramallah & Al-Bireh | Intervention |
|  | **Abu Falah** | Ramallah & Al-Bireh | Intervention |
|  | **Qarawah Bani Zeid** | Ramallah & Al-Bireh | Intervention |
|  | **Deir Amar** | Ramallah & Al-Bireh | Intervention |
|  | **Beituniya** | Ramallah & Al-Bireh | Intervention |
|  | **Beit Sera** | Ramallah & Al-Bireh | Intervention |
|  | **Deir Jarir** | Ramallah & Al-Bireh | Intervention |
|  | **Shuqba** | Ramallah & Al-Bireh | Intervention |
|  | **Beit rema** | Ramallah & Al-Bireh | Intervention |
|  | **Nea'leen** | Ramallah & Al-Bireh | Intervention |
|  | **Kharbatha Al-mesbah** | Ramallah & Al-Bireh | Intervention |
|  | **Qerah** | Salfit | Intervention |
|  | **Mardah** | Salfit | Intervention |
|  | **Farkha** | Salfit | Intervention |
|  | **Yasoof and Izkaka clinic** | Salfit | Intervention |
|  | **Sartah** | Salfit | Intervention |
|  | **Kefel Hares** | Salfit | Intervention |
|  | **Kufr Al-deel** | Salfit | Intervention |
|  | **Qarawah** | Salfit | Intervention |

*Clinic shut down by MoH in December 2016
